# Exposing the Molecular Screening Method of Indonesian Natural Products Derivate as Drug Candidates for Cervical Cancer

**Published:** 2017

**Authors:** Usman Sumo Friend Tambunan, Arli Aditya Parikesit, Mochammad Arfin Fardiansyah Nasution, Amalia Hapsari, Djati Kerami

**Affiliations:** a *Bioinformatics Research Group, Department of Chemistry, Faculty of Mathematics and Natural Science, Universitas Indonesia, Depok 16424, Indonesia. *; b *Mathematics Computation Research Group, Department of Mathematics, Faculty of Mathematics and Natural Science, Universitas Indonesia, Depok 16424, Indonesia.*

**Keywords:** Cervical cancer, Human papillomavirus (HPV), Indonesian natural products, HDAC, HDACi, CADDD approach, Herbaric acid

## Abstract

The menace of cervical cancer has reached an alarming rate. There are more than 450.000 cases of cervical cancer yearly, with mortality rate of about 50%. This deadly cancer is caused by human papillomavirus (HPV), mainly subtypes 16 and 18. The pharmaceutical industry has produced drug for combating the virus, known as SAHA (suberoylanilide hydroxamic acid). It inhibits class II HDAC *Homo sapiens *(HDACi). The utilization of SAHA has some side effects, one of which is bone loss. Thus, searching for viable alternatives aside SAHA is inevitable. The objective of this research is to investigate the molecular interaction of selected Indonesian natural products with class II HDAC *Homo sapiens.* LigX tool in MOE 2008.10 was used as an instrument to investigate the molecular interaction*. *Then, computer-aided drug discovery and development (CADDD) approach involving molecular docking and dynamics methods was utilized to screen the natural products library. In the end, we found that herbaric acid could act as a potential drug candidate for cervical cancer.

## Introduction

Indonesia is known as tropical forest country which recognized worldwide as one of the most biodiverse countries. There are around 25,000 species of flowering plants, the amount of which exceeds the other tropical areas in the world such as South America and West Africa. It exists, among others, medicinal plant species diversity (1). Based on the records of the WHO, IUCN, and WWF, there are more than 20,000 species of medicinal plants used by 80% of the population worldwide (2). Utilization of medicinal plants is expected to cure various diseases, such as cervical cancer (3).

Cervical cancer is formed in the cervical region, the organ that connects the uterus to the vagina. It is caused by the *Human papillomavirus* (HPV) (4). It is the second most common malignant tumor for women around the world and the leading cause of death from cancer for women in developing countries (5).

There are 11,150 new cases of cervical cancer and 50,000 cases of in situ carcinoma in the United States and more than 500,000 cases diagnosed worldwide each year (6). American Cancer Society (2013) predicts the number of new cases of invasive cervical cancer in the United States in 2008 reached 11,070 with 3,870 deaths. However, the number of new cases continues to decline each year. Epidemiological changes in the United States is due to the awareness of Pap smear early detection (8). However, the presence of cervical cancer continues to increase in developing countries. An estimated 40,000 cases of carcinoma of the cervix uteri occur in Indonesia every year (9, 10).

Some cancer treatments have been made​ with surgery, treatment, or chemotherapy (11). Until now, effective cancer drugs are undiscovered.This is due to the low selectivity of the drugs as well as the unknown process of carcinogenesis itself (12). Therefore, researchers are motivated to seek out the presence of more effective anti-cervical cancer drug. Scientists already have been working on HDAC inhibitors (HDACi), such as SAHA (Suberoylanilide hydroxamic acid) and TSA (Trichostatin A) as a remedy for cervical cancer (13). Currently, the trend is exposing the role of natural product compounds as HDACi (14). The computational tools are imperative in order to understand the working mechanism of HDACi and HDAC enzymes (15, 16).

Thus, the purposes of this research are to design ligand inhibitor class II HDAC *Homo sapiens* using natural product materials found in Indonesia; to analyze the results of molecular docking and molecular dynamics of ligand inhibitors of natural materials with class II HDACs; and also to test pharmacological properties, bioactivity, and ADMET (absorption, distribution, metabolism, and excretion – toxicity).

## Experimental

*Determination of the 3D structure of Class II HDAC Homo sapiens*

The pipeline for 3D structure determination was following our instead of the following r extended methods (17, 18). The sequence data were obtained from NCBI website (http://www.ncbi.nih.gov). The Multiple Sequence Alignment/MSA method was performed to the sequences obtained from NCBI website. The sequences in FASTA format were uploaded to the server of NCBI BLAST (Basic Local Alignment Search Tool) and Clustal Omega at EMBL-EBI, then BLASTP was performed. Then, the three-dimensional structure of the chosen class II HDAC *Homo sapiens* protein was obtained from Protein Data Bank (PDB) website (http://www.rcsb.org/pdb/home/home.do). If the PDB was not found on the server, the SWISS model online software (http://swissmodel.expasy.org/) was used for modeling the 3D protein structure (19). The retrieved structure will be used in the molecular docking analysis. Class II HDAC *Homo sapiens* utilized in this study were HDAC 4, 5, 6, 7, 9 and 10. Software used in the preparation stage was the MOE (Molecular Operating Environment) 2008.10 (20). The visualization of the 3D structure of the protein was generated by using Chimera 1.9 software (21).

*Design of Class II HDAC Inhibitor Ligands Homo sapiens*

In this study, ACDLabs ChemSketch 12.0 and MarvinSketch 17.2.13.0 software were used to design ligands for class II HDAC inhibitors *Homo sapiens*. The ligand has been designed to ACDLabs ChemSketch and MarvinSketch 17.2.13.0 software that can be further processed by the MOE software for the molecular docking and molecular dynamics (22).

*Preparation of ligand inhibitors of Class II HDAC Homo sapiens*

Ligand inhibitor used in this study was derived from natural product compounds in Indonesia that obtained from various sources (23-39).

*Molecular docking results analysis and complex interaction depict*

The validation method was following our pipeline (13). MOE 2008.10 was employed to perform molecular docking analysis. The simulation results obtained 100 best ligand positions based on the data of binding free energy (ΔG_binding_). The interaction between protein and ligand was analyzed. Rendering process was done to see the interaction of the ligand-enzyme complex in three-dimensions instead of three dimensions. MOE and Chimera software were used to illustrate the interaction of ligand inhibitor and the cavity of the enzyme in two-dimensional (2D) and three-dimensional (3D) structures, respectively.

*Screening drugs and drug analysis scan*

Using Lipinski’s Rule of Five and ADMET properties as a reference, drug scan was done to look at the feasibility of ligand inhibitor as cervical cancer drug candidates. In this phase, the drug scans were performed using Osiris Property Explorer, Toxtree v2.1.0, Lazar, FAF-Drugs2 and ACDLabs/I-Lab (40-43). Ligand screening was then conducted to obtain compounds with the best parameter values. ​

*Molecular dynamics simulation*

Molecular dynamics simulations were performed using MOE 2008.10 and GROMACS 4.6.5 (44, 45). The objective of the molecular dynamics simulation was to look at the dynamics of enzymes, ligands, and their interactions with the presence of a solvent. Generalized Born Implicit Solvent (GBIS) was used in the solvation mode with the AMBER approach when using MOE 2008.10, while GROMOS96 43a1 forcefield and SPC water model were used when performed using GROMACS 4.6.5 (46, 47). There are three stages in the molecular dynamics simulations, namely: initialization, equilibration, and production. These stages could be used to perform initialization timing, enzyme-ligand interactions in the solvent, and also stability curves based on RMSD (Root Mean Square Deviation).

## Results

*Sequence searching of Homo sapiens Class II HDAC*

**Figure 1:**
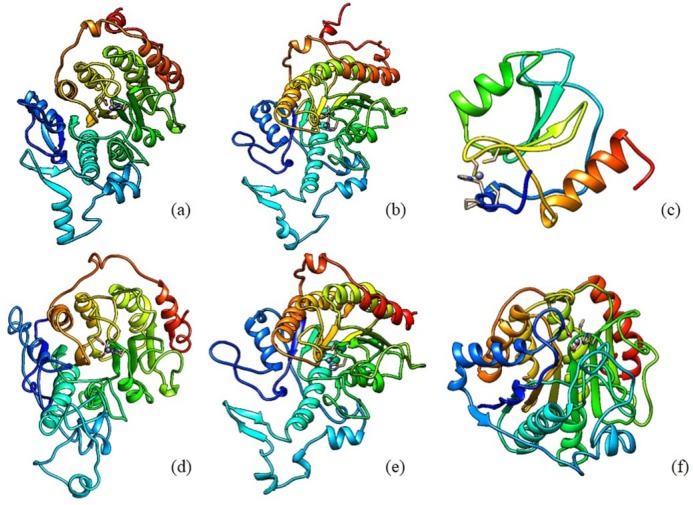
Structural models of HDAC Class II *Homo sapiens*: (a) HDAC 4 (b) HDAC 5 (c) HDAC 6 (d) HDAC 7 (e) HDAC 9 (f) HDAC 10

The discovery of class II HDAC *Homo sapiens* sequences was done by searching it at NCBI protein database. Class II HDAC *Homo sapiens* consists of HDAC 4, HDAC 5, HDAC 6, HDAC 7, HDAC 9, and HDAC 10. There are 65 types of protein sequence of those enzymes. The enzyme sequence selection based on several parameters, such as the suitability of long sequences, the use of enzyme sequences in various studies, and the novelty level of sequences. Sequences derived from the UniProt database KnowledgeBase (UniProtKB)/SWISS-PROT is a sequence that meets these parameters. The retrieved sequences for HDAC 4, HDAC 5, HDAC 6, HDAC 7, HDAC 9, and HDAC 10 are P56524.3, Q9UQL6.2, Q9UBN7.2, Q8WUI4.2, Q9UKV0.2, and Q969S8.1, respectively. The 3D structure of the retrieved HDAC sequences can be seen in Figure 1.

*Active site visualization of Class II HDAC Homo sapiens*

Catalytic site locations for each type of class II HDAC *Homo sapiens* are different. Determination of the active site could be made ​​from the results of the three-dimensional structure determination of HDAC 4, HDAC 6, and HDAC 7. 

As for HDAC 5, HDAC 9 and HDAC 10, the identification of the three-dimensional structure was derived from SWISS Model. LigX tool from MOE 2008.10 utilized to elucidate the interaction between the ligand inhibitors and the enzyme, in order to determine the active 

side. 

*Design of class II HDACi Homo sapiens*

The ligand design was done by using a wide variety of natural product compounds originated from Indonesia. ACDLabs was used to perform molecular compounds depiction. 

In this study, we were using SAHA, TSA, and valproic acid (VPA) as the standard ligand inhibitors against class II HDAC *Homo sapiens*. 

*Molecular docking simulation*

Molecular docking simulations were done for the interaction between the ligand with class II HDAC *Homo sapiens*. The interaction between ligand with the enzyme in the simulation is semi-flexible because the enzyme inhibitors are considered rigid, and the ligand is considered to be flexible. The software used in this simulation is MOE 2008.10. There are 2,020 ligand inhibitors used in these simulations, including three standard inhibitors, which are SAHA, TSA, and VPA. Repetitions performed during the simulation is 100 times, so the simulations contained 100 poses for each inhibitor where there were only be one of the best poses of each compound that was 

recorded. The result of molecular docking simulation is in the form Gibbs free energy values.

**Figure 2:**
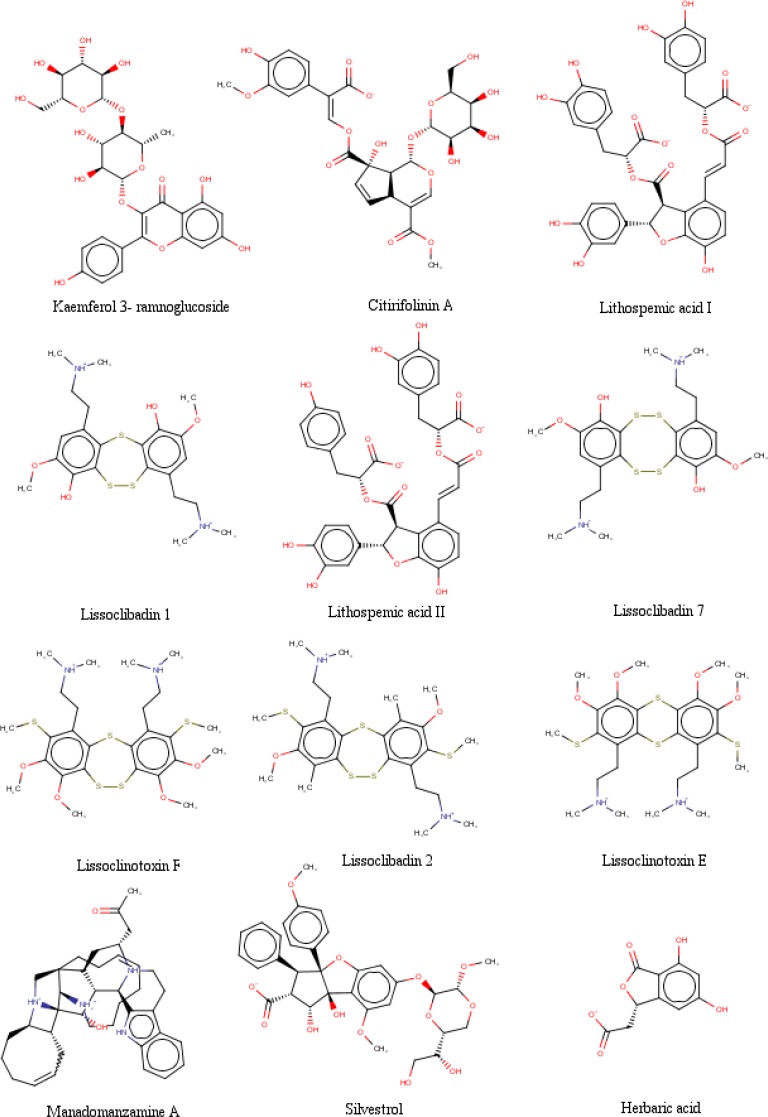
The chemical structures of selected Indonesian natural product compounds

**Figure 3:**
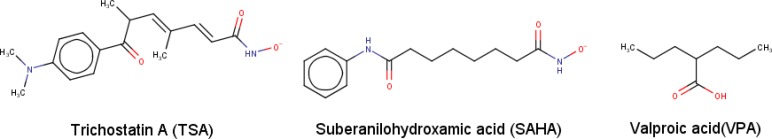
The chemical structures of the chosen standard ligands in this study

**Figure 4:**
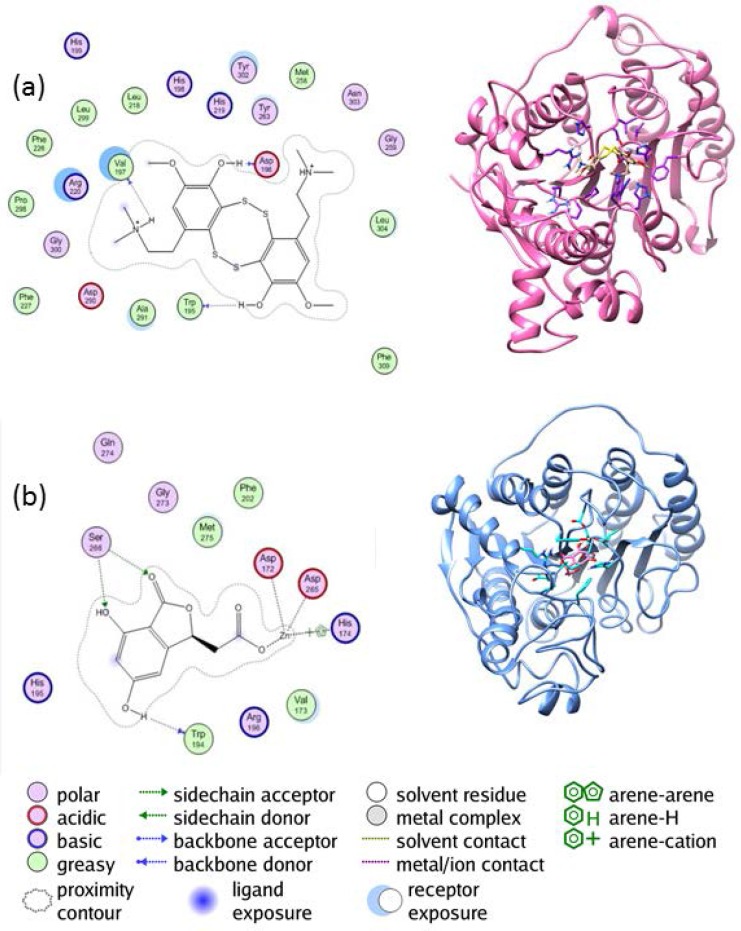
The interaction between (a) the enzyme HDAC4 and lissoclibadin 7 (b) the enzyme HDAC 10 and herbaric acid

**Figure 5:**
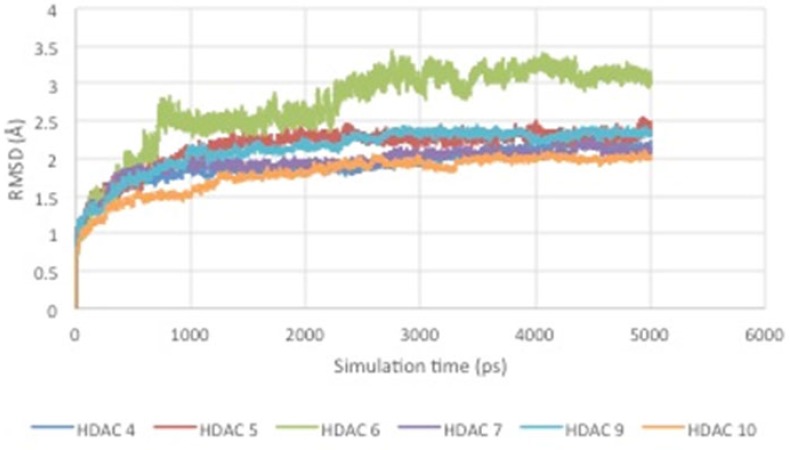
RMSD curve of HDAC enzyme-ligand complex.

**Figure 6:**
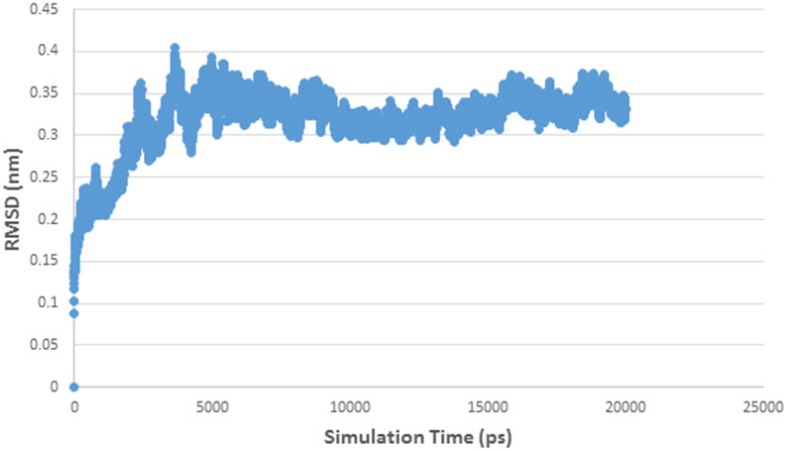
RMSD curve of HDAC 5 and herbaric acid complex

**Table 1:** *∆*G_binding _value of the twelve best and three standard ligands with the HDAC enzymes.

**No**	**Name**	**ΔG **_binding _ **(kJ/mol)**					
		**HDAC 4**	**HDAC 5**	**HDAC 6**	**HDAC 7**	**HDAC 9**	**HDAC 10**
1	Lithospemic acid II	-7.241	-12.883	-10.501	-12.618	-12.810	-9.720
2	Lithospemic acid I	-5.831	-9.401	-8.416	-9.499	-11.190	-6.404
3	Manadomanzamine A	-5.526	-7.351	-7.193	-12.094	-10.975	-8.712
4	Silvestrol	-3.001	-12.428	-9.407	-8.581	-8.772	-5.364
5	Herbaric acid	-7.136	-11.864	-7.781	-9.109	-7.198	-6.306
6	Lissoclibadin 7	-10.141	-7.141	-10.284	-10.168	-11.181	-7.256
7	Lissoclibadin 1	-10.110	-8.030	-9.506	-7.554	-11.284	-6.942
8	Citirifolinin A	-5.503	-6.348	-10.194	-11.434	-8.010	-4.996
9	Lissoclinotoxin F	-7.025	-11.048	-10.036	-10.938	-10.584	-5.235
10	Lissoclinotoxin E	-6.259	-6.541	-9.671	-10.876	-7.526	-8.948
11	Kaemferol 3- ramnoglucoside	-8.181	-8.159	-6.467	-8.812	-6.091	-10.915
12	Lissoclibadin 2	-7.562	-9.045	-5.495	-6.587	-8.207	-10.436
S1	SAHA	-5.030	-7.642	-5.743	-7.813	-4.957	-6.271
S2	TSA	-4.540	-8.315	-15.231	-6.568	-7.988	-5.349
S3	VPA	-4.609	-8.374	-9.230	-6.132	-8.243	-6.412

**Table 2.** inhibition constants value of the twelve best and three standard ligands with the HDAC enzymes

**No**	**Name**	_P_ **Ki**					
		**HDAC 4**	**HDAC 5**	**HDAC 6**	**HDAC 7**	**HDAC 9**	**HDAC 10**
1	Lithospemic acid II	5.276	9.386	7.651	9.193	9.333	7.082
2	Lithospemic acid I	4.248	6.849	6.131	6.920	8.153	4.665
3	Manadomanzamine A	4.026	5.356	5.240	8.811	7.996	6.347
4	Silvestrol	2.187	9.055	6.853	6.252	6.391	3.908
5	Herbaric acid	5.199	8.643	5.669	6.637	5.244	4.595
6	Lissoclibadin 7	7.388	5.203	7.493	7.408	8.146	5.286
7	Lissoclibadin 1	7.366	5.850	6.926	5.503	8.221	5.058
8	Citirifolinin A	4.009	4.625	7.427	8.330	5.836	3.640
9	Lissoclinotoxin F	5.118	8.049	7.312	7.969	7.711	3.814
10	Lissoclinotoxin E	4.560	4.765	7.046	7.924	5.483	6.519
11	Kaemferol 3- ramnoglucoside	5.960	5.944	4.712	6.420	4.438	7.952
12	Lissoclibadin 2	5.510	6.589	4.004	4.799	5.979	7.603
S1	SAHA	3.665	5.567	4.184	5.693	3.611	4.569
S2	TSA	3.307	6.058	11.097	4.785	5.819	3.897
S3	VPA	3.358	6.101	6.725	4.468	6.005	4.671

**Table 3:** The data of best ligands pharmacological properties according to Lipinski’s rules of five

**No**	**Name**	**Mw**	**Log P**	**TPSA**	**Rot. Bond**	**HBD**	**HBA**	**Violation**
1	Lithospemic acid II	702.61	4.34	257.81	14	8	15	4
2	Lithospemic acid II	716.61	3.98	278.04	14	9	16	6
3	Manadomanzamine A	608.86	5.15	71.60	2	3	6	2
4	Silvestrol	640.63	1.31	182.83	10	5	13	4
5	Herbaric acid	224.17	0.72	104.06	2	3	6	0
6	Lissoclibadin 7	514.74	4.04	166.60	8	2	6	1
7	Lissoclibadin 1	482.68	5.77	150.12	8	2	6	1
8	Citirifolinin A	610.52	-1.12	248.20	11	7	16	5
9	Lissoclinotoxin F	602.92	7.44	178.72	12	0	6	4
10	Lissoclinotoxin E	570.85	5.64	144.60	12	0	6	2
11	Kaemferol 3- ramnoglucoside	594.52	-0.38	249.20	6	9	15	4
12	Lissoclibadin 2	570.92	8.23	160.26	10	0	4	2
S1	SAHA	264.36	2.47	78.42	8	3	5	0
S2	TSA	302.37	2.68	69.64	6	2	5	0
S3	VPA	144.21	2.80	37.30	5	1	2	0

**Table 4:** Potential mutagenicity and carcinogenicity of the best ligands according to Benigni-Bossa rules

**No**	**Name**	**Potential Carcinogen Based on QSAR**	**Genotoxic Carcinogenicity**	**Nongenotoxic Carcinogenicity**	**Potential ** ***S. thyphimurium*** ** mutagenicity**
1	Lithospemic acid II	No	Negative	Negative	No
2	Lithospemic acid I	No	Negative	Negative	No
3	Manadomanzamine A	No	Negative	Negative	No
4	Silvestrol	No	Negative	Negative	No
5	Herbaric acid	No	Negative	Negative	No
6	Lissoclibadin 7	No	Negative	Negative	No
7	Lissoclibadin 1	No	Negative	Negative	No
8	Citirifolinin A	No	Negative	Negative	No
9	Lissoclinotoxin F	No	Negative	Negative	No
10	Lissoclinotoxin E	No	Negative	Negative	No
11	Kaemferol 3- ramnoglucoside	No	Negative	Negative	No
12	Lissoclibadin 2	No	Negative	Negative	No
S1	SAHA	No	Negative	Negative	No
S2	VPA	No	Positive	Negative	Yes
S3	TSA	No	Negative	Negative	No

**Table 5:** Prediction of ADMET properties based on ACD/I-Lab.

**No**	**Name**	**Probability Side Effect**						**Bioavailability**	
		**Blood**	**System cardiovascular**	**System gastrointestinal**	**Kidney**	**Liver**	**Lung**	**%F (Oral) >30%**	**%F (Oral) >70%**
1	Lithospemic acid II	0.74	0.96	1.00	0.98	0.86	0.90	0.033	0.008
2	Lithospemic acid I	0.74	0.96	1.00	0.98	0.86	0.90	0.033	0.008
3	Manadomanzamine A	0.94	1.00	1.00	0.99	0.99	0.97	0.290	0.039
4	Silvestrol	0.91	0.98	0.99	0.97	0.81	0.66	0.033	0.008
5	Herbaric acid	0.91	0.17	0.20	0.07	0.11	0.15	0.223	0.025
6	Lissoclibadin 7	0.59	0.95	0.99	0.38	0.07	0.92	0.060	0.025
7	Lissoclibadin 1	0.60	0.98	0.97	0.41	0.16	0.90	0.231	0.049
8	Citirifolinin A	1.00	0.99	0.95	0.78	0.98	0.92	0.033	0.008
9	Lissoclinotoxin F	0.32	0.64	0.96	0.57	0.05	0.53	0.231	0.039
10	Lissoclinotoxin E	0.33	0.81	0.95	0.61	0.13	0.47	0.231	0.039
11	Kaemferol 3- ramnoglucoside	0.94	0.94	0.98	0.38	0.67	0.74	0.033	0.009
12	Lissoclibadin 2	0.39	0.53	0.97	0.65	0.07	0.56	0.231	0.039
S1	SAHA	0.36	0.25	0.07	0.11	0.11	0.37	0.759	0.756
S2	TSA	0.59	0.50	0.51	0.27	0.52	0.63	0.909	0.432
S3	VPA	0.09	0.09	0.08	0.05	0.05	0.05	0.950	0.838

*Analysis of molecular docking simulation results*

*Determination of ligand inhibitor based on the best free energy and inhibition constants*

The data were obtained in the form of Gibbs free energy of binding (ΔG_binding_) between ligand inhibitors with HDAC enzymes. Ligand inhibitor that has the lowest ΔG_binding_ value is the best one. Three ligands have better ΔG_binding_ value than the standard ligands. They are lissoclibadin 7 that interacts with HDAC4 enzymes, litospermic II acid that interact with HDAC 5, 6, 7 and 9, and kaempferol 3-ramnoglucoside that interact with the enzyme HDAC10. Data of the best ΔG_binding_ can be seen in Table 1. Furthermore, the chemical structures of the best selected natural compound ligands and standard inhibitors can be seen in Figures 2 and 3, respectively (35,37 and 48-51).

In addition, the smaller the value of Ki, the larger tendency of the enzyme-ligand complex formation becomes. In other words, the enzyme-ligand complex formation is more feasible on the higher value of the pKi. Therefore, the value of Ki is used as one of the parameters of competitive inhibition ability between the ligands with others. The most competitive ligand inhibitor is the one with the largest pKi value. Table 2 shows the best ligands inhibition constant value at 300 K.

From Table 2, it can be seen that lissoclibadin 7 has the highest pKi value for HDAC 4 inhibitor compared to the others. Lithospermic acid II tendency to form the enzyme-ligand complex occurs when the ligand interacts with HDAC5, HDAC6, HDAC7 and HDAC9. Moreover, there are also kaempferol-3-ramnoglucoside that has the best ability of the enzyme inhibition of HDAC 10.

The interaction between the enzyme HDAC4 and lissoclibadin 7 can be observed from Figure 4(a). There are interactions between lissoclibadin 7 with amino acid residues contained in the charge-relay system, Asp196 and Val197. It occurred in the form of hydrogen bonds and a proton donor. Furthermore, there are also interactions between the hydroxyl group of amino acid residues Trp195 to form hydrogen bond. Interactions that occur at amino acid residues Trp195, Asp196 and Val197, occurred at its backbone. There is no interaction between lissoclibadin 7 with Zn^2+^ as cofactor ligand inhibitors that are already interacting with other amino acid residues. However, Figure 4(b) shows different results. It is clear that Asp172, Asp265, and His174 interacted with the Zn^2+^ cofactor. In this end, the role of the metal cofactor was much more clearly exposed in the herbaric acid compared to lissoclibadin 7.

*Screening of ligand inhibitor compounds as the best natural ingredients*

*Based on the nature of pharmacology*

Predictions of pharmacological properties aswere employed based on Lipinski’s rule of five. They were performed using online software ACD/I-Lab and FAF-Drugs2. Results of the pharmacological properties of ligand prediction are presented in Table 3.

The analysis of the pharmacological properties from the table above was based on several parameters. The first parameter is molecular weight (MW), where it should be between 160 to 500 daltons. Lissoclibadin 1 and herbaric acid are compounds that escaped from these parameters, while the lithospermic acid I, lithospermic acid II, manadomanzamine A, silvestrol, lissoclibadin 7, citrifolinin A, lissoclinotoxin F, lissoclinotoxin E, kaempferol 3-ramnoglucoside and lissoclibadin 2 have a molecular weight in above 500 daltons.

The second parameter is log P, where P is logarithmic of octanol-water partition coefficient that indicates the polar property of compounds. The reference values for log P is -0.4 to +5.6. Four ligands violate these parameters, which are lissoclibadin I, citrifolinin A, lissoclinotoxin F, and lissoclibadin 2, while for the lithospermat acid II, lithospermic acid I, manadomanzamine A, silvestrol, herbaric acid, lissoclibadin 7, lissoclinotoxin E and kaempferol 3-ramnoglucoside are between the reference values. The third parameter is the TPSA (Topological Polar Surface Area) which showed the extent of the surface of the inclined polar compounds. The extent of TPSA should not be more than 140 Å because if it is too broad, the ligand will be easily removed from the body through the urine. 

The fourth parameter is Rotational Bond. It is related to the ligands rigidity. The fifth and sixth parameters are the Hydrogen Bond Donor and Acceptor in which the amount must be below or equal to 10. All ligands escape the Hydrogen Bond Donor parameters. As for the Hydrogen Bond Acceptor, there are four ligands that violated it, which are lithospermic acid I, silvestrol, citrifolinin A and kaempferol 3-ramnoglucoside. Ligands that do not violate the Hydrogen Bond Acceptor are lithospermic acid II, manadomanzamine A, herbaric acid, lissoclibadin 7, lissoclibadin 1, lissoclinotoxin F, lissoclinotoxin E and lissoclibadin 2. It can be concluded that the best ligand is herbaric acid because it does not violate any parameters from Lipinski’s Rule of Five.

*Based on benigni-bossa mutagenicity and carcinogenicity*

After conducting screening based on pharmacological properties, further testing of mutagenicity and carcinogenicity potential have been carried out in accordance with the Benigni-Bossa rules. This test is based on the existence of clusters of fragments of the mutagenic or carcinogenic chemical.

From the results of toxicity testing using Toxtree v2.1.6 software, Table 4 showed that all ligand inhibitors have mutagenic and carcinogenic properties. Genotoxicity and carcinogenicity occur because of irreversible genetic damage to the DNA structure. Non-genotoxic carcinogenesis does not affect DNA directly but induces cancer through other processes, such as modulation of certain hormones or proteins. Also, all the best ligand does not have the potential mutagens to *Salmonella typhimurium* TA100 based-Ames test and the potential carcinogens based on QSAR (41).

Through the analysis of the above data, it can be concluded that all the ligand inhibitors, as well as the standard inhibitors, SAHA, and VPA, have the mutagenicity and carcinogenicity property. Furthermore, the best ligand has safer genotoxic and carcinogenic mutagenic potential towards *S. typhimurium* than the standard, TSA.

*Based on health effects and bioavailability*

From the ligand inhibitors that have passed the Benigni-Bossa rule test, then the prediction of health effects and compound bioavailability were conducted. Predictions of these characteristic were done by using the online software ACD/I-Lab. The data obtained from the test results can be seen in Table 5.

Based on the Table 5, it can be seen that a wide variety of ligand inhibitors have certain health effects, especially toward blood, cardiovascular system, digestive system, kidneys, liver, and lung. The closer the parameters value to zero, then the lower the health effects. In addition, the highest threshold value that can be tolerated is 0.85. Red indicates a potent hazardous threat to health, while the green color indicates that the compound is relatively safe for health. It turned out that almost all of the ligand inhibitors have bad effects on the body, except herbaric acid. Moreover, all ligand inhibitors have a low bioavailability when consumed orally because its bioavailability value is below 30%.

*Molecular dynamics simulation analysis*

After analyzing the results of ligand inhibitor screening based QSAR and ADMET, the selected ligands, herbaric acid compounds, would undergo molecular dynamics simulation. The herbaric acid was chosen because it does not violate any parameter of Lipinski’s Rule of Five, does not have the potential of carcinogenicity and mutagenicity, has very low possible negative health effects on the body. It still has the potential to be drug lead even though its oral bioavailability is low.

After the screening phase was done, molecular dynamics simulation was then performed. Flexibility and dynamics are essential protein characteristics in the process of substrate recognition and molecules inhibition that can be described by the biophysical models of induced fit (53). The preparation of enzyme-ligand complex was set to Amber99 forcefield and Generalized Born Implicit Solvent (GBIS) solvent modes. The simulation was carried out at a temperature of 27 °C/300 K to room temperature in order to resemble the *in-vitro* conditions and 37 °C/310 K in order to resemble the normal human body temperature as a cervical cancer patient for *in-vivo* conditions. Furthermore, the settings were made ​​to resemble the atmosphere on the surface of the earth that is equal to 1 atm. There are three stages to be traversed in molecular dynamics simulations, the initialization phase, equilibration, and production.

*Analysis of molecular dynamics simulations*

From the results of molecular dynamics simulations, it can be seen that there is a conformational change of the enzyme-ligand complex due to the influence of temperature and solvent. In addition, the RMSD curve over the time can be seen in Figure 5.

Figure 5 shows RMSD stability of the enzyme-ligand complex at a specific time frame. The HDAC4-herbaric acid complex was stabilized at 2,25 Å, HDAC5-herbarat acid at 3 Å, HDAC6-herbaric acid at 3 Å, HDAC7-herbaric acid at 2 Å, HDAC9-herbaric acid at 2.4 Å, and HDAC10-herbaric acid at 2 Å. Then stability of the HDAC 5 and herbaric acid can be observed further by using GROMACS 4.6.5 in 20 ns (20.000 ps) molecular dynamics simulation (45). 

Figure 6 shows the HDAC 5–herbaric acid complex was stabilized at 3.2 Å. This result was not much different from the previous simulation results. During this period, the enzyme-ligand conformation is considered to have favorable stability. Therefore, herbaric acid can be developed as a lead compound for the treatment of cervical cancer.

## Discussion

Based on our previous research, the pipeline for the lead compound determination of HDAC class II inhibitor is evolving on the modification of the universally-available semi-synthetic compounds (17, 18 and 54). However, some groups already employed Indonesian natural products to computationally evaluate their feasibility as lead compounds (55–57). It should be noted that none of those groups use molecular dynamics and ADME-Tox methods to comprehend the structural stability of enzyme-ligand interaction and its pharmacological properties. Hence, in order to optimize the utilization of Indonesia natural products, the existing pipeline, that already included molecular dynamics and ADME-Tox methods, was applied to this research (58, 59). 

Our virtual screening pipeline has found herbaric acid as the most favorable lead compound (60–62). The origin of herbaric acid is unique, as it was found from the metabolites of Indonesian fungal species (25). Thus, the function of herbaric acid is still unknown, but it shows bioactivity at brine shrimp assay (25). The methods to synthesize herbaric acid in the lab are Heteroatom-Directed Reverse Wacker Oxidations and Sonogashira/oxacyclisation copper(I) process (63, 64). Thus, it is possible to synthesize and test the activity of herbaric acid in the lab. At the end, utilization of HDAC class II-based bioassay to determine the bioactivity of herbaric acid should be possible (65). 

Although novel approaches to drug design, such as fragment and structural based design, are being developed, the natural products lead compounds library still deemed as a cardinal choice for pushing the novel drug candidates (66–70). However, due to its extreme diversity of structural types, synthesizing natural product compounds will always be a daunting challenge for a chemist (71–74). Before applying the synthetic laboratory method, it is better to use bioinformatics tool to obtain insight of the synthetic accessibility of the respective compounds (75–77).

## Conclusion

Several conclusions can be drawn from this research. First, there are some of the best ligand inhibitors which have better binding free energy values compared to the standard. This suggests that the interaction between the enzyme-ligand are preferred compared to the standard. In addition, there are ligand inhibitors which have better pKi value compared to the standard. This indicates that the inhibitor has stronger tendency to form ligand-ligand complex than the standard. The analysis of enzyme-ligand interactions shows that the standard ligand compounds and ligands of the natural products form an interaction with Zn^2+ ^cofactor in the same binding site. The residues which interact with Zn^2+^ cofactor are the oxygen atom in the cluster of C = O and -OH. However, some ligand inhibitors do not bind directly to the Zn^2+ ^cofactor but interacts with amino acid residues in its vicinity and works by blocking the path leading to the cofactor Zn^2+^.

Some conclusions were also obtained from the results of ligand inhibitor screening based on several parameters. Based on Lipinski’s Rule of Five, only herbaric acid does not violate one of its parameters. While screening based on Benigni-Bossa, none of which has the potential ligand inhibitors of carcinogenesis or mutagenesis properties. 

The screening was based on the ADMET and their health effects, where only herbaric acid was deemed safe for health, despite its low bioavailability when taken orally. Therefore, herbaric acid is the only ligand that undergoes the molecular dynamics simulations. It shows that herbaric acid has good stability in the enzyme-ligand complex. Thus, herbaric acid could be developed as a lead compound for the treatment of cervical cancer. 
